# Supplementary material for: Sharing of heteroplasmies between human liver lobes varies across the mtDNA genome
Source: Sci Rep. 2019 Aug 2;9:11219. doi: 10.1038/s41598-019-47570-1 (PMC6677727; doi:10.1038/s41598-019-47570-1)
Supplement: Supplementary file 1 — Supplementary figures [file 41598_2019_47570_MOESM1_ESM.pdf]

Sharing of heteroplasmies between human liver lobes varies across the mtDNA genome

Alexander Hübner[§], Manja Wachsmuth[§], Roland Schröder, Mingkun Li, Anna Maria Eis-Hübinger, Burkhard Madea and Mark Stoneking

[§] These authors contributed equally to the manuscript.

Supplementary Figures

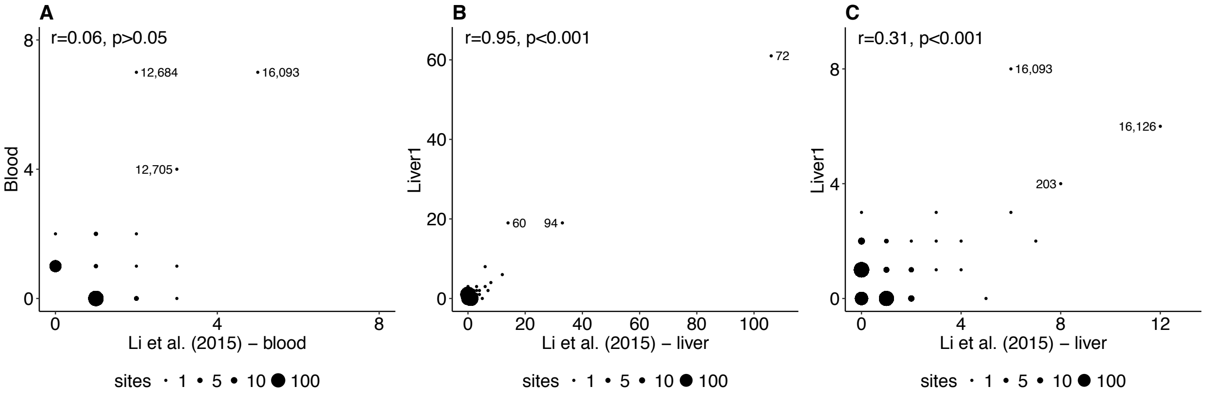

Supplementary Figure 1: Comparison of heteroplasmic sites found in this study with a previous study (Li et al. 2015). Number of heteroplasmic individuals for each site identified in this study is compared to that from a previous study (Li et al. 2015). The size of a dot indicates how many sites are plotted at the same position in the scatter plot. Comparison for **A** blood samples; **B** liver samples; **C** liver samples, without sites 60, 72, 94.

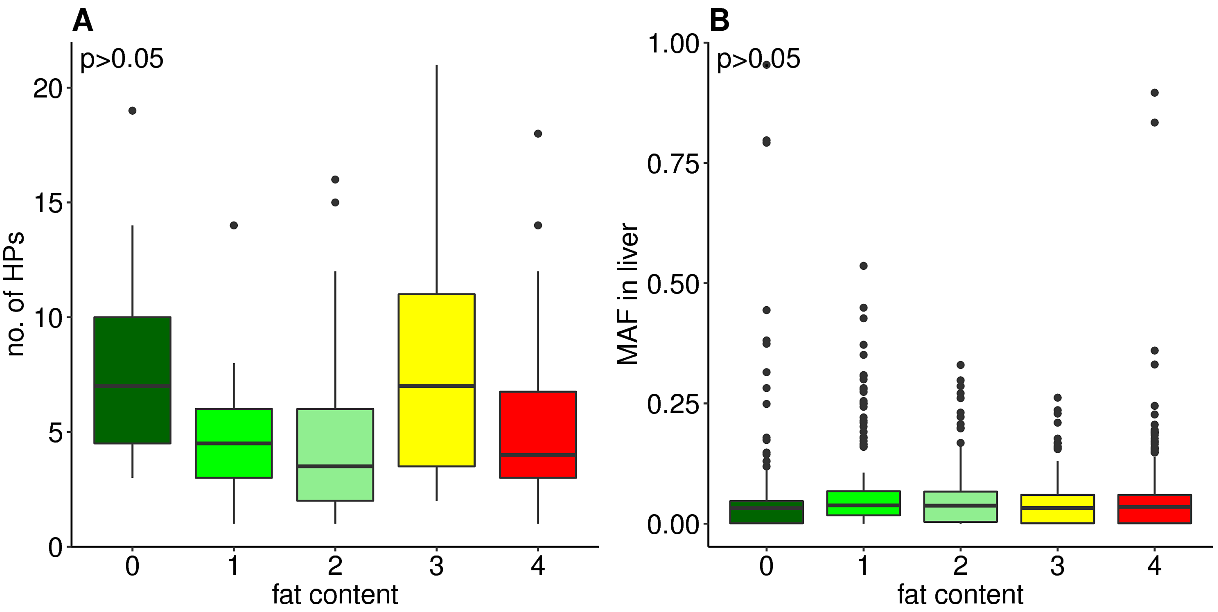

Supplementary Figure 2: Liver fat content and heteroplasmy. For all fat content groups (no fat (0), low fat (1), medium fat (2), high fat (3) and adipohepatic (4)), the **A** total number of heteroplasmic sites and **B** MAF at single sites are shown. P-values for the Mann-Whitney U test comparing group 0 and group 4 are given.

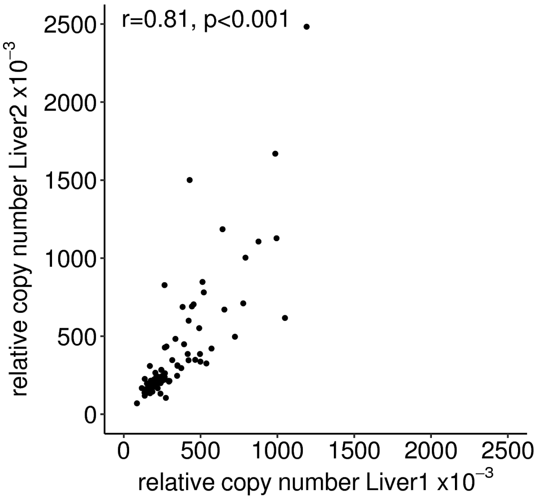

Supplementary Figure 3: Relative mtDNA copy number. Relative copy number, determined as described previously (Wachsmuth et al. 2016) in liver sample 1 versus sample 2. Pearson's correlation coefficient r is given.

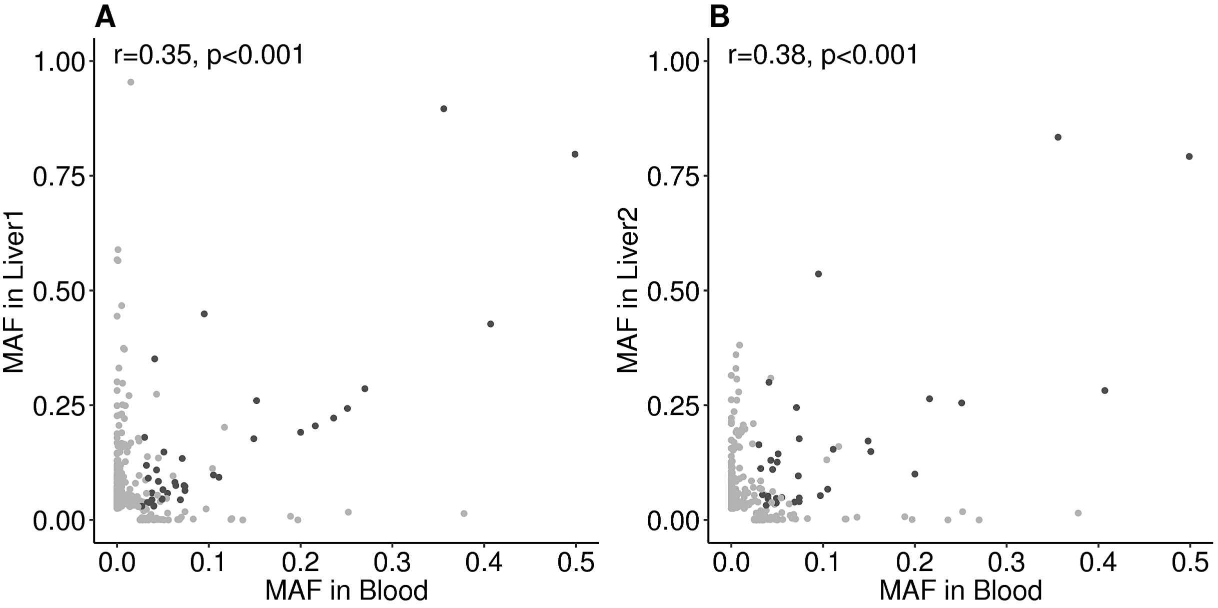

Supplementary Figure 4: Correlation of MAFs at heteroplasmic sites in blood and liver. Each dot is one heteroplasmic site in one individual. Pearson's correlation coefficient r is given. Heteroplasmic sites are compared in **A** blood and liver sample 1, **B** blood and liver sample 2. Dark grey dots indicate sites that were shared (i.e., heteroplasmic in both blood and the respective liver sample from an individual), light grey dots indicate sites that were not shared.

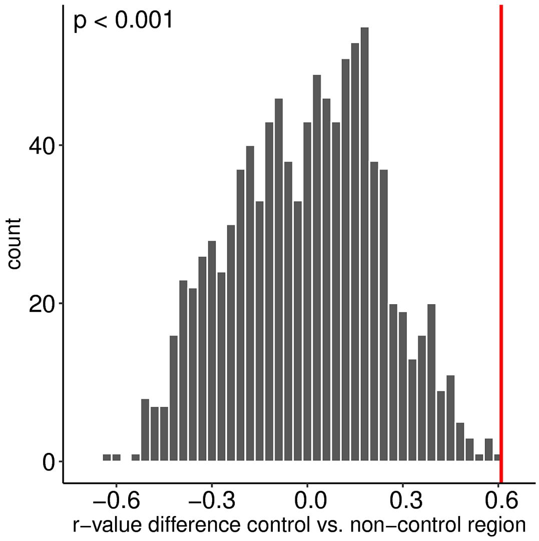

Supplementary Figure 5: Distribution of r-value differences for random partitions of all heteroplasmic sites into two sets, one with the same number of sites as the control region (210 sites) and one with the same number of sites as the non-control region (281 sites). The red bar indicates the observed difference between the r-values for the control region and the non-control region.

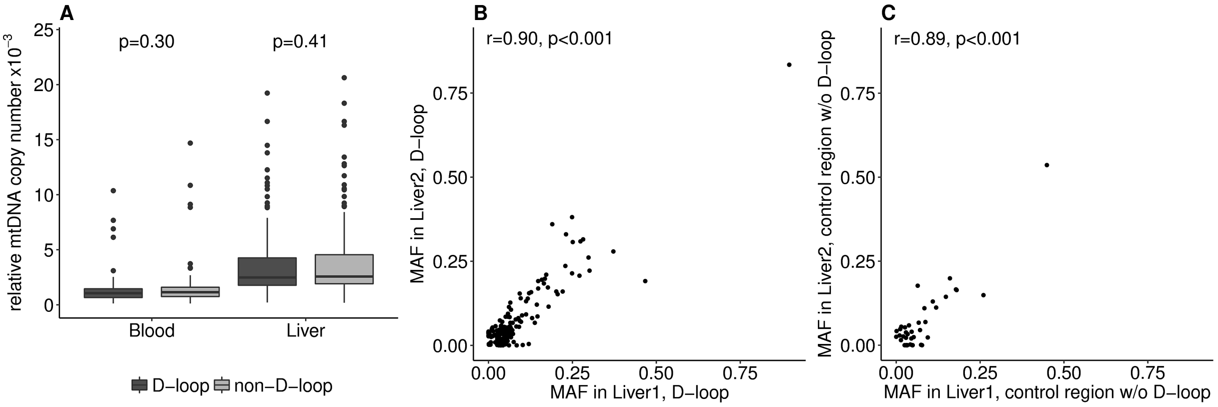

Supplementary Figure 6: Heteroplasmy sharing in liver samples and correlation with 7S DNA. A distribution of relative mtDNA copy numbers per mtDNA region (D-loop (dark grey) vs. non-D-loop (light grey)) and tissue. P-value for the Mann-Whitney U test comparing D-loop and non-D-loop is given. **B-C** The MAFs at heteroplasmic sites are compared for: **B** sites in the D-loop region; and **C** sites in the control region but outside the D-loop region. Each dot is one heteroplasmic site in one individual. Pearson's correlation coefficient r is given.

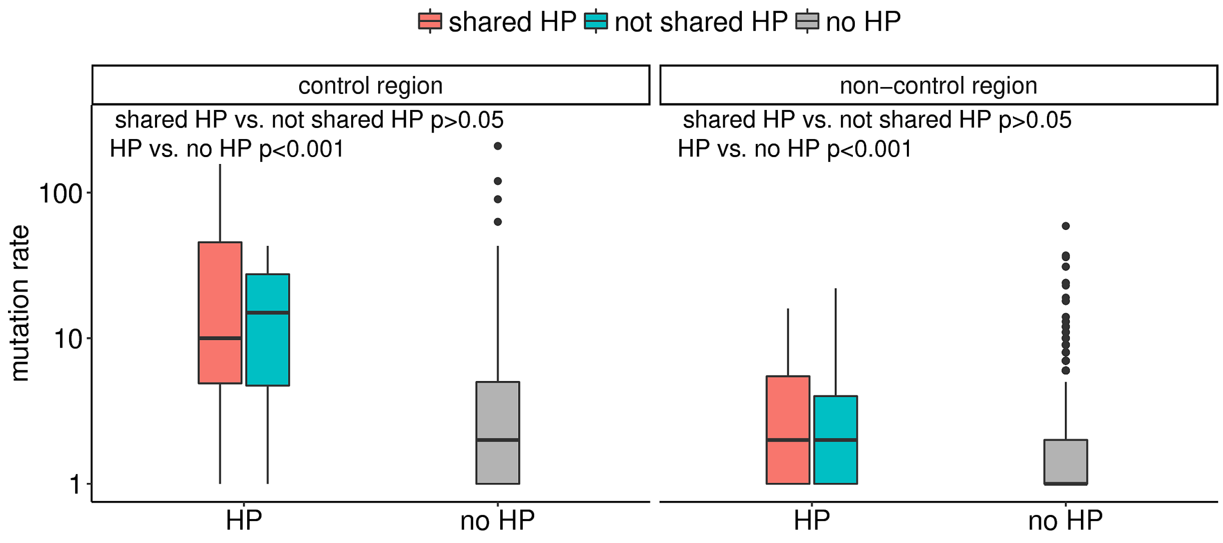

Supplementary Figure 7: Mutation rate and heteroplasmy sharing. Average estimated mutation rate (Soares et al. 2009) for heteroplasmic sites (HP) that are shared vs. not shared vs. sites that were not heteroplasmic, separately for within and outside the control region. Mann-Whitney U test was done for shared vs. non-shared heteroplasmies and for heteroplasmies vs. sites that were not heteroplasmic. Only polymorphic sites (i.e. those with reported mutation rates > 0 (Soares et al. 2009)) were included.

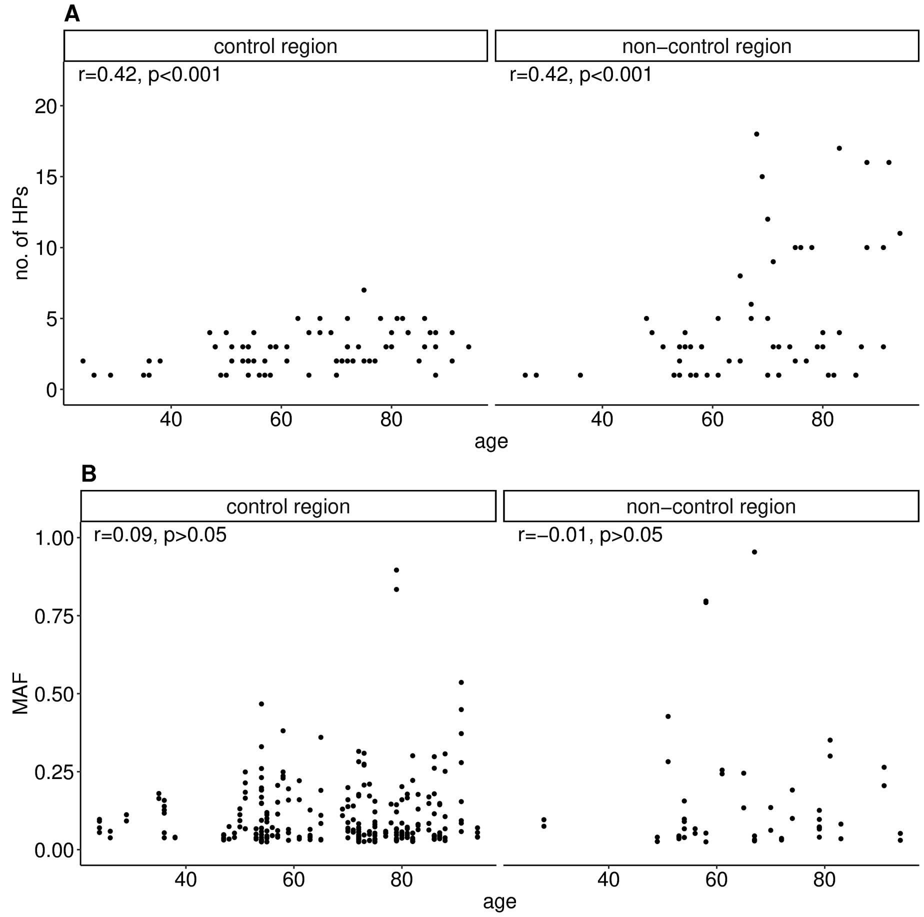

Supplementary Figure 8: Heteroplasmy and age. A Total number of heteroplasmic sites per individual (sum of liver sample 1 and 2) versus age separately for within and outside of the control region. **B** MAF versus age separately for within and outside of the control region. Each dot is one heteroplasmic site in one sample.

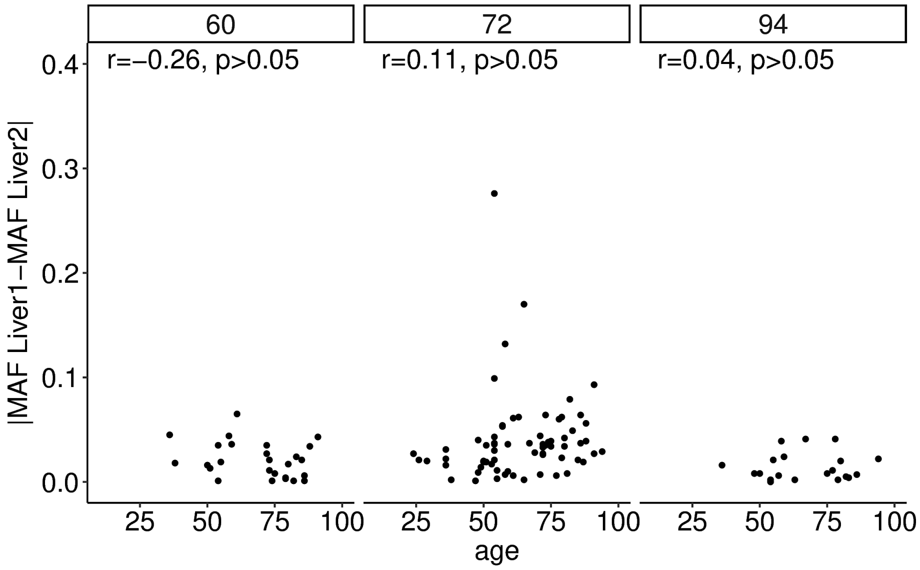

Supplementary Figure 9: MAF and age correlation for sites 60, 72 and 94. Each dot is one heteroplasmic site in one individual. Pearson's correlation coefficient r is given.

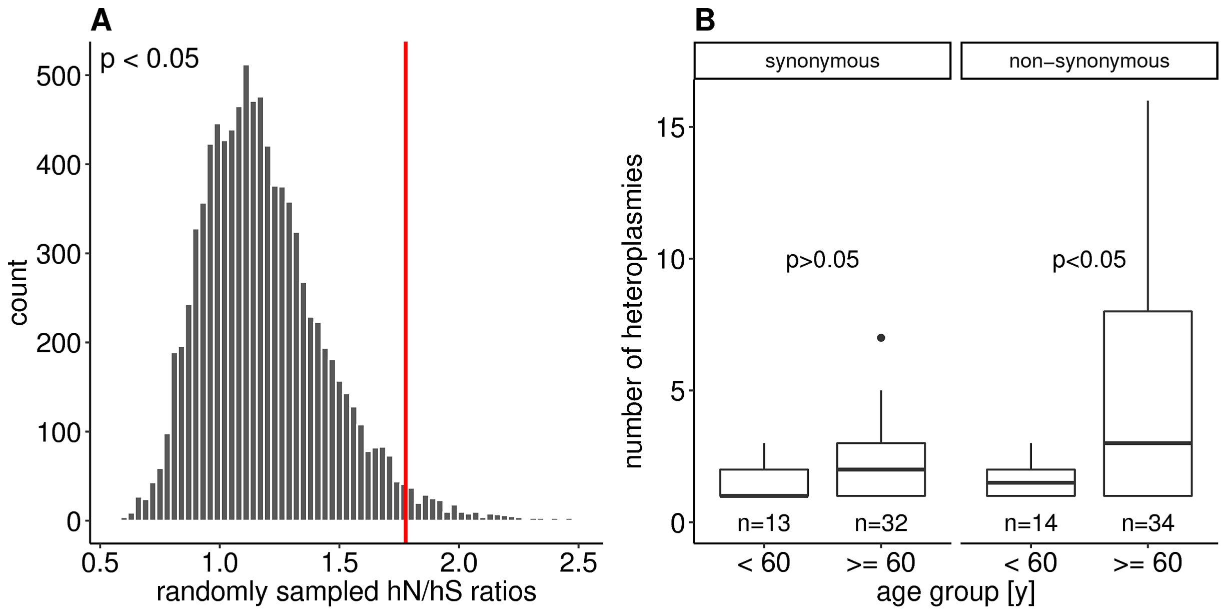

Supplementary Figure 10: Excess of non-synonymous vs. synonymous heteroplasmies. A Significance tests for the ratio of non-synonymous heteroplasmies per non-synonymous site vs. synonymous heteroplasmies per synonymous site. The red vertical bar indicates the observed hN/hS ratio for liver specific heteroplasmic sites. Distribution of 10,000 hN/hS-ratios calculated by comparing the rCRS to itself after introducing the mutations observed for liver specific heteroplasmies at random sites across the coding region. **B** The distribution of the number of synonymous and non-synonymous heteroplasmies within age groups < 60 years and >= 60 ages. Mann-Whitney U test was done for age group >= 60 years vs. age group < 60 years separately for synonymous and non-synonymous heteroplasmies. The p-value was adjusted for multiple testing.

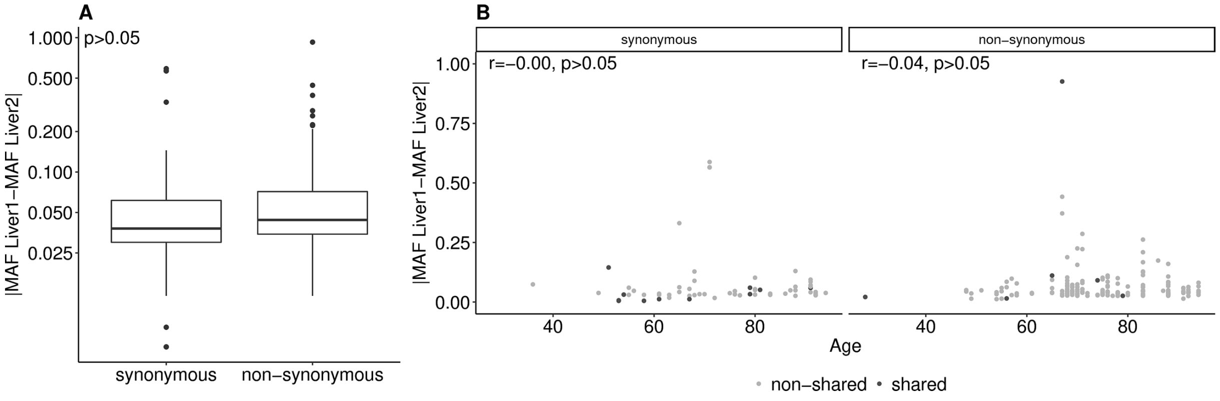

Supplementary Figure 11: MAF difference in the coding region with age. A Distribution of difference in MAF between corresponding liver samples for synonymous and non-synonymous heteroplasmies. The p-value for the Mann-Whitney U test comparing the distribution of synonymous and non-synonymous heteroplasmies is given. The y-axis is plotted in log-scale. **B** correlation of the difference in MAF with age plotted for synonymous and non-synonymous heteroplasmies separately. Dark grey dots indicate sites that were shared (i.e., heteroplasmic in both blood and the respective liver sample from an individual), light grey dots indicate sites that were not shared. Each dot is one heteroplasmic site in one individual. Pearson's correlation coefficient r is given for both subsets.

References

Li M, Schroeder R, Ni S, Madea B, Stoneking M. 2015. Extensive tissue-related and allele-related mtDNA heteroplasmy suggests positive selection for somatic mutations. *Proceedings of the National Academy of Sciences of the United States of America* **112**: 2491-2496.

Soares P, Ermini L, Thomson N, Mormina M, Rito T, Rohl A, Salas A, Oppenheimer S, Macaulay V, Richards MB. 2009. Correcting for Purifying Selection: An Improved Human Mitochondrial Molecular Clock. *American Journal of Human Genetics* **84**: 740-759.

Wachsmuth M, Huebner A, Li M, Madea B, Stoneking M. 2016. Age-Related and Heteroplasmy-Related Variation in Human mtDNA Copy Number. *Plos Genetics* **12**: e1005939.
